# Epithelial N-cadherin and nuclear β-catenin are up-regulated during early development of human lung

**DOI:** 10.1186/1471-213X-10-113

**Published:** 2010-11-16

**Authors:** Riitta Kaarteenaho, Elisa Lappi-Blanco, Siri Lehtonen

**Affiliations:** 1 Institute of Clinical Medicine, Department of Internal Medicine/Respiratory Research Unit, Centre of Excellence in Research, University of Oulu and Oulu University Hospital, Oulu, Finland; 2 Institute of Diagnostics, Department of Pathology, University of Oulu and Oulu University Hospital, Oulu, Finland; 3 PO.Box 22, FI-90029 Oulu University Hospital, Finland; 4 Clinical Research Center, Oulu University Hospital, Oulu, Finland; 5 Institute of Clinical Medicine, Department of Surgery, University of Oulu and Oulu University Hospital, Oulu, Finland; 6 PO.Box 5000, FI-90014 University of Oulu, Finland

## Abstract

**Background:**

The aim of this study was to analyze the cell-specific expression of E- and N-cadherin and β-catenin in developing human lung tissues from 12 to 40 weeks of gestation.

**Methods:**

Fortyseven cases of developing human lung including pseudoglandular, canalicular, saccular and alveolar periods were analyzed by immunohistochemisty for E- and N-cadherin and β-catenin and twentyone cases were also investigated by RT-PCR for E- and N-cadherin and β-catenin. For identifying the lung cells, the sections were also stained with antibodies against thyroid transcription factor-1 (TTF-1) and caveolin-1. Normal adult lung tissue was used as a control.

E-cadherin was strongly expressed in epithelium of bronchi and large bronchioles from week 12 onwards and it was also positive in alveoli in pretype II cells and type II cells. N-cadherin was present in most of the epithelial cells of bronchi and the largest bronchioles during the pseudo-glandular and canalicular periods. N-cadherin was not detected in epithelium of developing alveoli. β-catenin was strongly membrane-bound and positively expressed in bronchial epithelium from week 12 to week 40; it showed nuclear positivity in both developing airway epithelium and in the cells underneath the epithelium during pseudo-glandular period and to a lesser degree also in the canalicular period. β-catenin was positive in pretype II cells as well as in type I and type II pneumocytes within alveoli.

RT-PCR analyses revealed detectable amounts of RNAs of E- and N-cadherin and β-catenin in all cases studied. The amounts of RNAs were higher in early stages of gestation.

**Conclusions:**

E-cadherin is widely expressed in bronchial and alveolar epithelial cells. N-cadherin exhibit extensive epithelial positivity in bronchial epithelial cells during early lung development. The presence of β-catenin was observed in several cell types with a distinct location in tissue and cells in various gestational stages, indicating that it possesses several roles during lung development. The expressions of protein and mRNAs of E- and N-cadherin and β-catenin were higher in early gestation compared to of the end. Moreover, the expressions of these factors were higher during the lung development than in the adult human lung.

## Background

There is still little detailed knowledge of how cells differentiate during ontogenesis in the lung as well as in pulmonary diseases. It has been assumed that signalling processes occuring during pulmonary fibrosis and lung cancer may share similarities with the various stages of human lung development, thus by studying lung development it might be possible to acquire valuable information that may be useful when researching fibrotic and neoplastic lung diseases [1]. The epithelial changes are regulated by the extracellular matrix (ECM), which is an essential element in the process of branching morphogenesis, and also participating in the control of cell phenotype expression. In our previous studies, we have observed that the levels of several ECM proteins e.g. tenascin-C and precursors of collagen I and III are increased in certain localizations during human lung development as well as in fibrotic lung disorders when compared to the healthy adult normal lung [2-5].

Cells are connected with each other or the ECM in different ways but it is clear that adhesion molecules are extremely important in cellular junctions. The cadherins represent an important subclass of adhesion molecules [6]. The cytoplasmic domain of cadherins interacts with catenins, and this complex associates with actin filaments [7]. The cadherin superfamily consists of several members and one of these, E-cadherin is expressed in epithelial cells and it is nowadays a commonly used marker of cell epithelial phenotype in studies focusing on the epithelial-mesenchymal transition (EMT) [8]. N-cadherin was originally found to be expressed in neural and muscle cells, but subsequently it is observed be an element of mesenchymal cells [9]. In studies investigating the EMT, N-cadherin has been used as a marker of mesenchymal differentiation [8]. Specific cadherins have been shown to directly stimulate cellular differentiation into certain types of tissue [10]. Recently, expression of N-cadherin has been observed to be present in epithelial lung tumors in addition of E-cadherin and β-catenin [11].

β-catenin serves many roles in the maintenance of cell architecture, for example it can bind to the cytoplasmic tail of E-cadherin. The WNT/β-catenin signal transduction pathway has been shown to control embryonic patterning [12]. The cell specific expression profile of E- and N-cadherin during developing human lung is still unclear, though the role of β-catenin in lung organogenesis has been evaluated more extensively [13]. A previous study indicated that β-catenin signalling was required for the formation of the distal, but not the proximal, airways, and that the excision of β-catenin in epithelial cells caused respiratory failure and death [14].

The aim of the immunohistochemical study was to examine the cell-specific expression of E- and N-cadherin and β-catenin in normal human developing lung at different gestational ages i.e. from week 12 to week 40 during the pseudoglandular, canalicular, saccular and alveolar periods. We hypothesized that the expression and localization of these factors could vary during the different developmental periods. In addition to protein localization by immunohistochemistry, the amounts of RNAs were evaluated in some cases by quantitative real-time reverse transcriptase polymerase chain reaction (RT-PCR).

## Methods

### Patients and handling of specimens

Samples of lung tissue were retrieved from the files of the Department of Pathology, Oulu University Hospital. The study protocol was approved by the Ethical committee of the Northern Ostrobothnia Hospital District (Stament 64/2001, amedment 68/2005). The National Supervisory Authority for Welfare and Health (Valvira) has given a license for this study for the use of lung tissues of abortive foetuses for research purposes (Reg. nr. 7323/05.01.00.06/2009). Since this is a retrospective material, the informed consent permission has been given by the National Supervisory Authority for Welfare and Health, which is the national licensing authority for social welfare and health care (please see http://www.valvira.fi/en/licensing).

The study material for developing lung consisted of 47 cases of spontaneous abortion, stillbirth, and autopsied infants who had died for different reasons without lung disorders within 1-2 days after birth in Oulu University Hospital between 1990 and 2002. Autopsies had been performed within one day in most cases and within two days in four cases. Causes of death of the infants were abortion (n = 19), abruption of placenta (n = 9), rupture of fetal membranes (n = 2), feto-fetal transfusion (n = 2), sacral teratoma (n = 2), prolapse or aplasia of the umbilical artery (n = 3), placentitis or chorioamnionitis (n = 4), hemochromatosis (n = 1), hydrocephalus (n = 1), meningomyelocele (n = 1), encephalocele (n = 1), holoprosencephaly (n = 1) and hemorrhage of caput (n = 1). Infants with pneumonia, cardiac abnormalities or features of maceration were excluded. The gestational ages of infants ranged from 12 to 40 weeks, corresponding to the pseudoglandular (day 52 to week 16, 13 cases), canalicular (weeks 16-28, 17 cases), saccular (weeks 28-36, 9 cases) and alveolar (weeks 36-40, 8 cases) periods. Uninvolved peripheral lung tissue from adults, used as a control, was obtained from six patients operated on for benign lung tumour. The clinical information was obtained from the patient records.

Lung samples, which had been taken from different parts of the left or right lung were fixed in 10% formalin and then dehydrated and embedded in paraffin. Sections, which were 5 μm thick, were stained with hematoxylin-eosin. All material was re-evaluated, and one representative tissue block from each case was selected for the immunohistochemical studies. In order to identify the phenotype of the various lung cells, the sections were also stained with antibodies against thyroid transcription factor (TTF-1) (a staining for type II pneumocytes) and caveolin-1 (a staining for type I pneumocytes) and some selected cases also with vimentin (a staining for fibroblast and myofibroblast).

### Antibodies and immunohistochemical staining

E-cadherin antibody (clone HECD-1) and N-cadherin antibody (clone 3B9) were purchased from Zymed Laboratories Inc (South San Francisco, CA, USA). β-catenin antibody (clone 5) was purchased from Transduction Laboratories (Lexington, KY, USA), TTF-1 (clone 8G7G31) from Dako (Carpinteria, CA), caveolin-1 (clone E249) from Abcam (Cambridge, UK) and vimentin (clone V9) from DakoCytomatin (Glostrup, Denmark). The sections were incubated overnight with antibodies to E-cadherin (diluted as supplied 1: 300), N-cadherin (diluted as supplied 1: 200), β-catenin (diluted as supplied 1:200), vimentin (diluted as supplied 1:1500), TTF-1 (diluted as supplied 1:100) and caveolin-1 (diluted as supplied 1:250). Immunohistochemistry was performed using paraffin-embedded biopsies. Tissue samples were sectioned and deparaffinized followed by an antigen retrieval procedure suitable for the antibody in question. Dako Envision Kit (Dako) was used in the immunohistochemistry with a 30 min incubation for the primary antibody. Negative controls were obtained by substituting non-immune rabbit or mouse serum and PBS for the primary antibodies.

### Scoring of the immunoreactivity

The extent and intensity of E- and N-cadherin and β-catenin were evaluated semiquantitatively as negative (0), weak (+), moderate (++) or strong (+++) in different types of pulmonary cells, such as epithelial cells of bronchioles and bronchus, alveolar epithelium including pretype II cells, type I and II pneumocytes, endothelial cells, interstitial cells such as fibroblasts and myofibroblasts, and mesothelial cells. In the evaluation, membrane-bound positivity, cytoplasmic and nuclear positivity were quantified.

### Quantitative real-time reverse transcriptase polymerase chain reaction (RT-PCR)

In 21 cases representing all developmental periods (pseudoglandular, n = 4; canalicular, n = 8; saccular, n = 7; alveolar, n = 4) one tissue block was selected for E-cadherin, N-cadherin and β-catenin analysis at the transcriptional level by quantitative real-time reverse transcriptase polymerase chain reaction (qRT-PCR) as previously described [15]. Additionally five paraffin embedded samples of normal adult human lung were used as controls. As it has been shown previously RNA can be isolated from paraffin embedded tissue material for expression profiling [16,17]. The total RNA was isolated from five 10 μm thick slices from each sample with Purelink FFPE total RNA isolation kit according to the manufacturer's instructions (Invitrogen, Carlsbad, CA, USA). The isolated RNA was quantified and qualified spectrophotometrically and 0.5 μg of isolated RNA was converted in duplicate to cDNA by RevertAid first strand cDNA synthesis kit (Fermentas, EU) by using random hexamers as primers. Quantitative RT-PCR was performed by using the iQ5 Optical system (Bio-Rad Laboratories, Hercules, CA) with SYBR Green I technology (iQ Custom SYBR green supermix, Bio-Rad Laboratories). PCR was performed in duplicate from each cDNA by using 66°C as the annealing temperature. The primers were 5'-aaggtgacagagcctctggat-3'(forward, f) and 5'-cgtctgtggctgtgacct-3'(reverse, r) for E cadherin; 5'-cgagccgcctgcgctgccac-3 (f) and 5'-cgctgctctccgctccccgc-3 (r) for N-cadherin; 5'-tggatgggctgcctccaggtgac-3' (f) and 5'-accagcccacccctcgagccc-3'(r) for β-catenin; and 5'-gagtcaacggatttggtcgt-3' (f) and 5'-gacaagcttcccgttctcag-3'(r) for glyceraldehyde-3-phosphate dehydrogenase (GAPDH) that was considered as a house-keeping gene in the data analysis. The results were counted by the Livak ΔΔC_t _method using a sample of normal adult lung as a reference [18]. Differences between the groups were analysed by PASW Statistics software version 17 (SPSS, Chicago, IL, USA) by using Kruskal-Wallis test.

## Results

### Immunohistochemistry for E- and N-cadherin and β-catenin

#### Pseudoglandular period (weeks 12 to 16)

**Figure 1:**
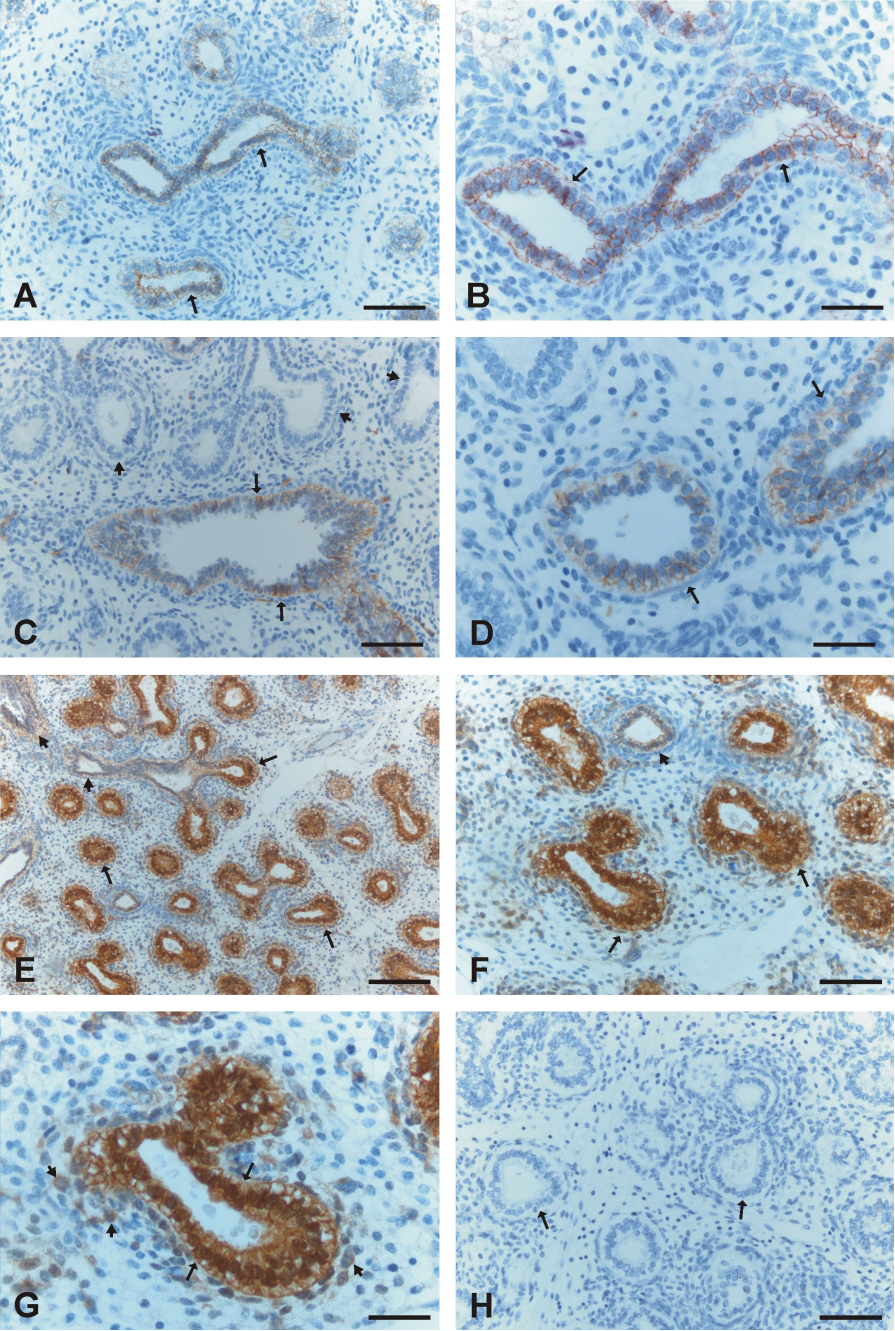
**Immunohistochemical stainings for E- and N-cadherin and β-catenin during pseudoglandular period of developing human lung**. 1A and 1B. Epithelium of the developing airways is positive for E-cadherin (arrows). 1C and 1 D. Epithelium of some developing airways is positive for N-cadherin (arrows) whereas other developing airways are negative for N-cadherin (short arrows). 1E and 1F. Epithelial and mesenchymal cells of developing airways are positive for β-catenin. In most airways epithelial cells display nuclear positivity (arrows) whereas some airways exhibit membrane-bound positivity (short arrows). 1G. A high power field image showing nuclear positivity for β-catenin in epithelial cells of a small developing airway (arrows) surrounded by positively stained mesenchymal cells (short arrows). 1 H. A negative control in which the primary antibody has been substituted with non-immune serum. Arrows are indicating developing airways. Scale bars = 40 μm in 1A, 1C, 1F and 1H; scale bars = 20 μm in 1B, 1 D and 1G, and scale bar = 80 μm in 1E.

E-cadherin was strongly expressed in all the epithelial cells of developing bronchi and bronchioles of all sizes including also the smallest structures (Figure 1A, 1B). N-cadherin was also moderately or strongly expressed in the epithelium of bronchi and largest bronchioles whereas it was negative in the smallest developing airways (Figure 1C, 1D). β-catenin displayed strong membrane-bound positivity in bronchi and larger bronchioles, whereas its expression in the small developing airways was mainly strongly nuclear (Figure 1E, 1F, 1G). The cells underneath the epithelium, especially in smallest airways, showed also nuclear positivity for β-catenin (Figure 1G), and in contrast, these non-epithelial cells at the same localizations seemed to be negative for E-and N-cadherin.

#### Canalicular period (weeks 16 to 28)

**Figure 2:**
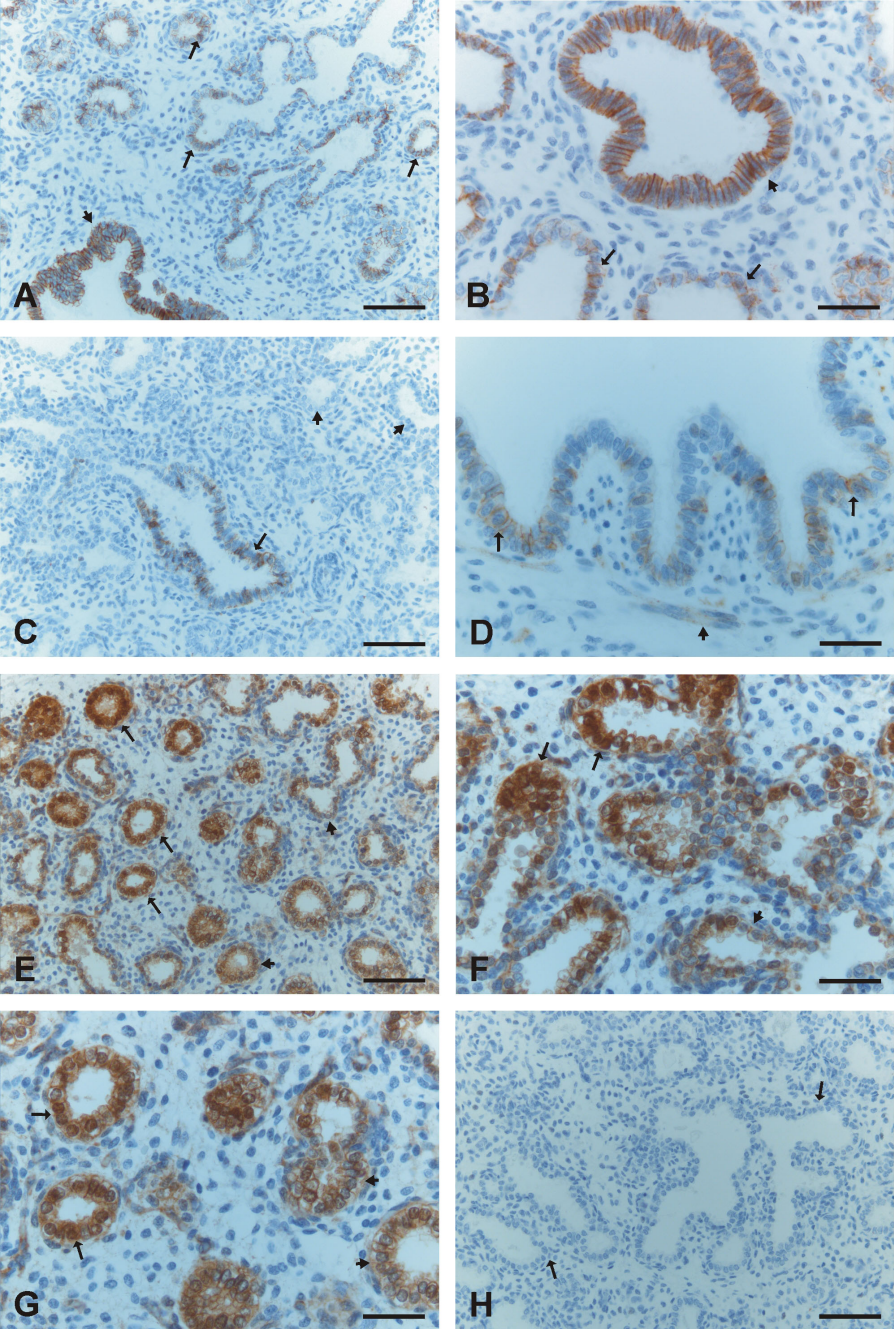
**Immunohistochemical stainings for E- and N-cadherin and β-catenin during canalicular period of developing human lung**. 2A and 2B. Epithelial cells of a developing bronchiolus (short arrows) and alveoli including pretype II pneumocytes (arrows) are positive for E-cadherin. 2C. Pretype II pneumocytes (short arrows) of alveoli are negative for N-cadherin while epithelial cells of a bronchiolus (arrow) are positive for N-cadherin. 2 D. In addition of the epithelial cells (arrows), some spindle shaped cells of a bronchiole showed also a positivity for N-cadherin (short arrow). 2E, 2F and 2G. Epithelial cells of developing airways including pretype II pneumocytes are positive for β-catenin showing mostly a nuclear positivity (arrows) and also a membrane-bound positivity in some cells (short arrows). 1 H. A negative control in which the primary antibody has been substituted with non-immune serum. Scale bars = 40 μm in 2A, 2C, 2E and 2 H; scale bars = 20 μm in 2B, 2D, 2F and 2G.

E-cadherin was strongly expressed in the epithelial cells of bronchi and bronchioles and also in the pretype II cells of developing alveoli (Figure 2A, 2B). N-cadherin was faintly or moderately expressed in the cells of bronchi and bronchioles whereas the cells of developing alveoli were negative (Figure 2C, 2D). β-catenin was strongly membrane-bound positive in the cells of bronchi and bronchioles, whereas the cells of alveoli showed mainly nuclear-type of positivity especially at the beginning of the stage (Figure 2E, 2F, 2G). However, at the end of this period, the amount of membrane-bound positivity within alveolar epithelium increased, and correspondingly, the nuclear positivity decreased.

#### Saccular (weeks 28 to 36) period

**Figure 3:**
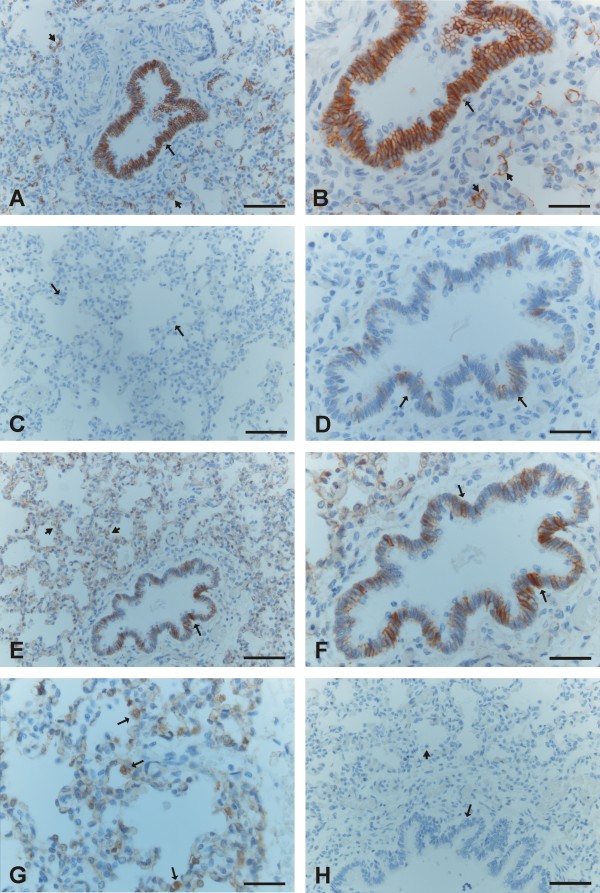
**Immunohistochemical stainings for E- and N-cadherin and β-catenin during saccular period of developing human lung**. 3A and 3B. A strong positivity for E-cadherin is seen in the epithelial cells of a bronchiole (arrows) and type II pneumocytes of alveoli are also positive for E-cadherin (short arrows). 3C. Alveolar epithelium is negative for N-cadherin (arrows). 3 D. A few epithelial cells of a bronchiole are positive for N-cadherin (arrows). 3E and 3F. Positive staining for β-catenin is seen in the epithelial cells of a bronchiole (arrows) and alveolar epithelial cells (short arrows). 3G. A high power field image showing a positive expression for β-catenin within alveoli. Some alveolar epithelial cells exhibit nuclear staining (arrows). 3 H. A negative control in which the primary antibody has been substituted with non-immune serum. Scale bars = 40 μm in 3A, 3C, 3E and 3H; scale bars = 20 μm in 3B, 3D, 3F and 3G.

E-cadherin was expressed strongly in the epithelial cells of bronchi and bronchioles with its expression being clearly positive within alveoli in type II cells (Figure 3A, 3B). N-cadherin was positive in some of the epithelial cells in bronchi and bronchioles, but its expression in alveoli remained negative (Figure 3C, 3D). β-catenin revealed membrane-bound positivity in the cells of bronchi and bronchioles, and the cells of alveolar epithelium were also mainly membrane-type positive, although some alveolar epithelial cells exhibited also nuclear-type positivity (Figure 3E, 3F, 3G).

#### Alveolar (weeks 36 to 40) period

**Figure 4:**
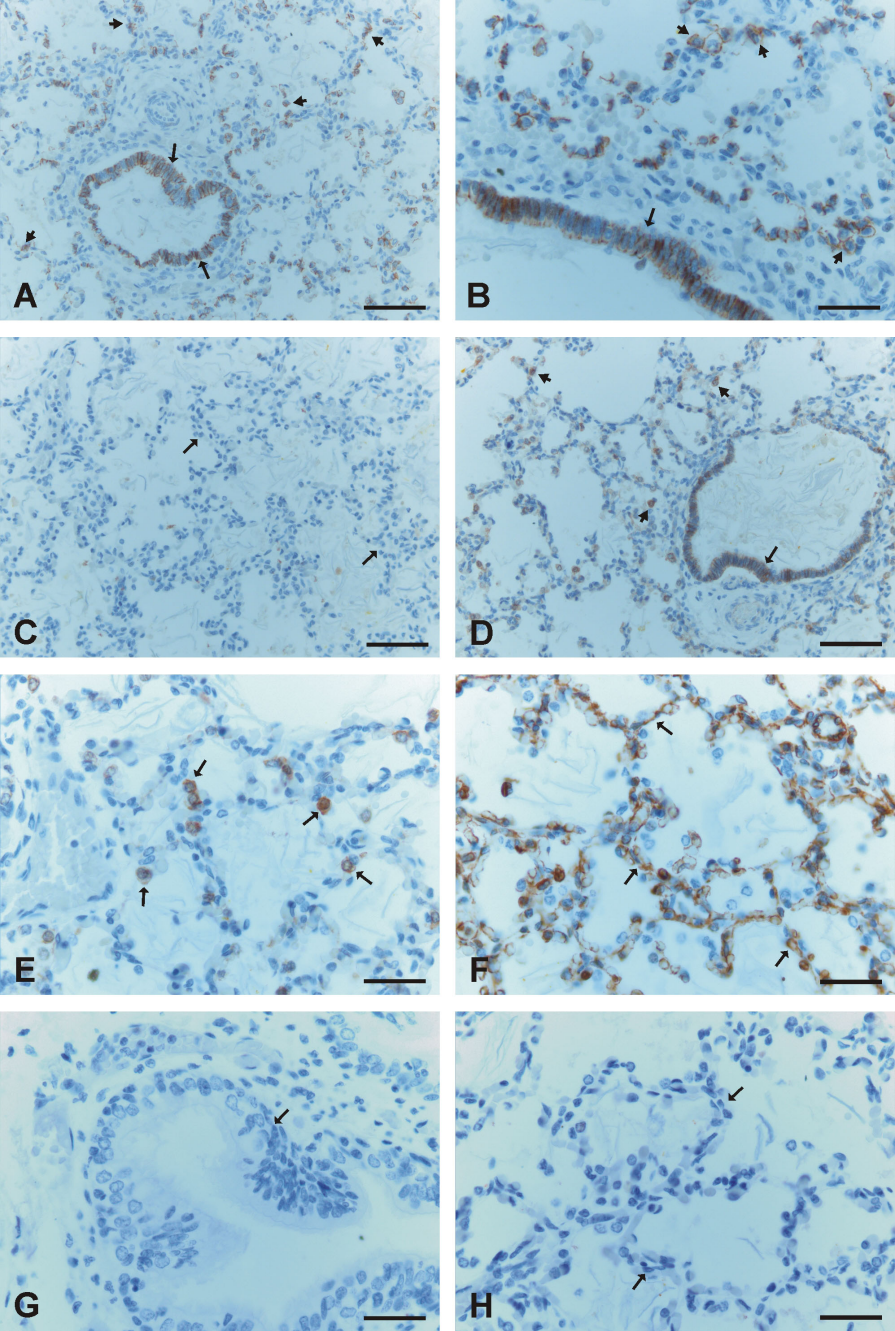
**Immunohistochemical stainings for E- and N-cadherin and β-catenin during alveolar period of developing human lung**. 4A and 4B. Type II pneumocytes (short arrows) and bronchial epithelial cells (arrows) display a positive staining for E-cadherin. 4C. Alveoli do not show any positivity for N-cadherin. 4 D. Type II pneumocytes of alveoli (arrows) are strongly positive for β-catenin. 4F. An example of vimentin staining showing that alveoli are positive for it. 4G. A negative control in which the primary antibody has been substituted with non-immune serum. 4 H. A negative control in which the primary antibody has been substituted with PBS with haematoxylin counterstain. Scale bars = 40 μm in 4A, 4C and 4D; scale bars = 20 μm in 4B, 4E, 4F, 4G and 4 H.

E-cadherin was moderately positive in the epithelium of bronchi and bronchioles and in type II pneumocytes within alveoli (Figure 4A, 4B). N-cadherin was faintly expressed in only a few scattered cells of the epithelium of bronchi and bronchioles. Most of the bronchial epithelial cells were negative for N-cadherin, nor could it be detected in the alveoli (Figure 4C). β-catenin revealed strong membrane-bound positivity in the cells of bronchi and bronchioles, and the cells of alveolar epithelium were also mainly membrane-type positive, although some alveolar epithelial cells showed also nuclear-type positivity (Figure 4D, 4E).

Mesothelium, fibroblasts, myofibroblasts, smooth muscle cells and endothelium were positive for β-catenin, but negative for E- and N-cadherin. The neural cells displayed positivity for N-cadherin. In some cases during the pseudoglandular period, trachea was included within the tissue sample, and its epithelium was positive for E- and N-cadherin and β-catenin. Negative controls obtained by substituting non-immune rabbit or mouse serum and PBS for the primary antibodies showed no positive staining (Figure 1H, 2H, 3H, 4G, 4H).

The results of the scoring of the immunoreactivity for various proteins are shown in Table 1. One score shown for each protein in various periods is valid for every case analyzed. If the samples of the same developmental stage showed variable staining with each other, two scores are shown.

**Table 1:** Immunoreactivity scores for E- and N-cadherin, β-catenin, TTF-1 and caveolin-1 in different types of pulmonary cells in developing human lung during various gestational periods.

	Pseudo-glandular period day 52-week 16 (13 cases)	Canalicular period weeks 16-28 (17 cases)	Saccular period weeks 28-36 (9 cases)	Alveolar period weeks 36-40 (8 cases)
**E-cadherin**				
bronchus	+++	+++	+++	++
bronchiolus	+++	+++	+++	++
alveolus	# +	* + or ++	# +	# +
endothelial cell	negative	negative	negative	negative
stromal cells	negative	negative	negative	negative

**N-cadherin**				
bronchus	++	++	+	(+)
bronchiolus	+ or ++	++	+	(+)
alveolus	# negative or +	negative	negative	negative
endothelial cell	negative	negative	negative	negative
stromal cells	negative	negative	negative	negative

**β-catenin**				
bronchus	+++ m	+++ m	+++ m	+++ m
bronchiolus	+++ m	+++ m	+++ m	+++ m
alveolus	# +++ n	* +++ n and m	#++ m and (n)	# ++ m and (n)
endothelial cell	+	+	+	+
stromal cells	+++ n	+ n	negative	negative

**TTF-1**				
bronchus	+	+	+	+
bronchiolus	++	+ or ++	+	+
alveolus	# +++	* +++	# +	# +
endothelial cell	negative	negative	negative	negative
stromal cells	negative	negative	negative	negative

**Caveolin-1**	negative	negative	negative	negative
bronchus	negative	negative	negative	negative
bronchiolus	negative	negative	+++	+++
alveolus	+	+	+	+
endothelial cell stromal cells	+	+	+	+

# small developing airways, not alveoli: * pretype II cells: # type II pneumocytes

m = membrane-bound positivity; n = nuclear positivity

#### Normal adult lung as a control

**Figure 5:**
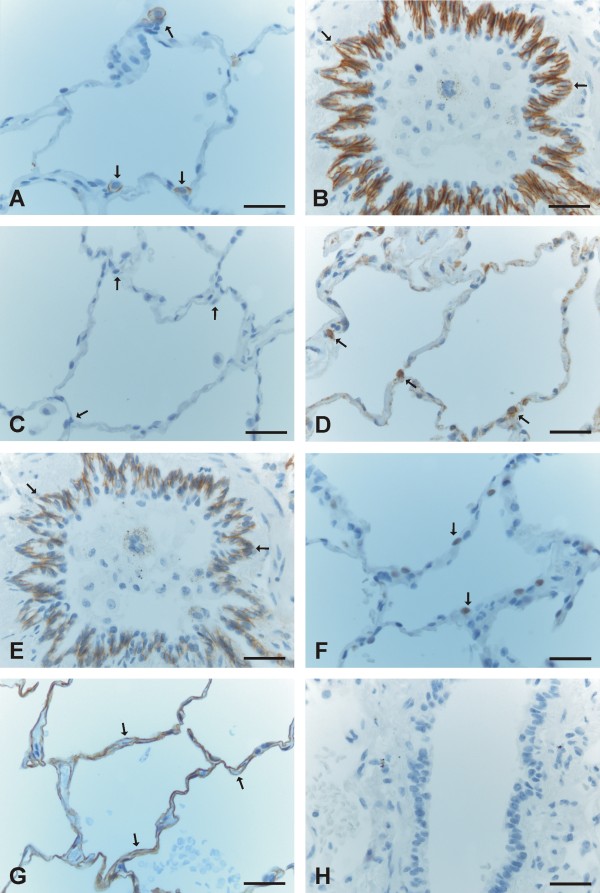
**Immunohistochemical stainings for E- and N-cadherin, β-catenin, TTF-1 and caveolin-1 in adult human lung**. 5A. Type II pneumocytes of alveoli are positive for E-cadherin (arrows). 5B. Epithelial cells of a bronchiole are expressing E-cadherin (arrows). 5C. Cells of alveoli are negative for N-cadherin (arrows). 5 D. Type II pneumocytes (arrows) and to a lesser extent also type I pneumocytes of alveoli are positive for β-catenin. 5E. Epithelial cells of a bronchiole are positive for β-catenin. 5F. Type II pneumocytes exhibit a positive staining for TTF-1 (arrows). 5G. A staining of caveolin-1 is positive in alveolar epithelium (arrows). 5G. A negative control in which the primary antibody has been substituted with non-immune serum. Scale bars = 20 μm.

E-cadherin was expressed in most epithelial cells of normal adult human lung including type II pneumocytes and also in all bronchial epithelial cells whereas mesothelial cells remained negative (Figure 5A). Alveolar epithelial cells including both type I and II pneumocytes were negative for N-cadherin (Figure 5B), while mesothelial cells and some scattered basal cells of bronchioles were positive. β-catenin was expressed in type II pneumocytes and epithelium of bronchioles and also faintly in type I pneumocytes (Figure 5C, 5D). Endothelial cells, some mesenchymal cells, and mesothelium stained also positively for β-catenin. The staining pattern for β-catenin was mainly membranous, but some type II pneumocytes exhibited cytoplasmic expression pattern for β-catenin with nuclear enhancement. Type II pneumocytes and some epithelial cells of bronchioles showed a positive staining for TTF-1 (Figure 5E). Cells lining alveoli expressed positively for caveolin-1 (Figure 5F).

### Immunohistochemistry for TTF-1 and caveolin-1

#### Pseudoglandular period (weeks 12 to 16)

**Figure 6:**
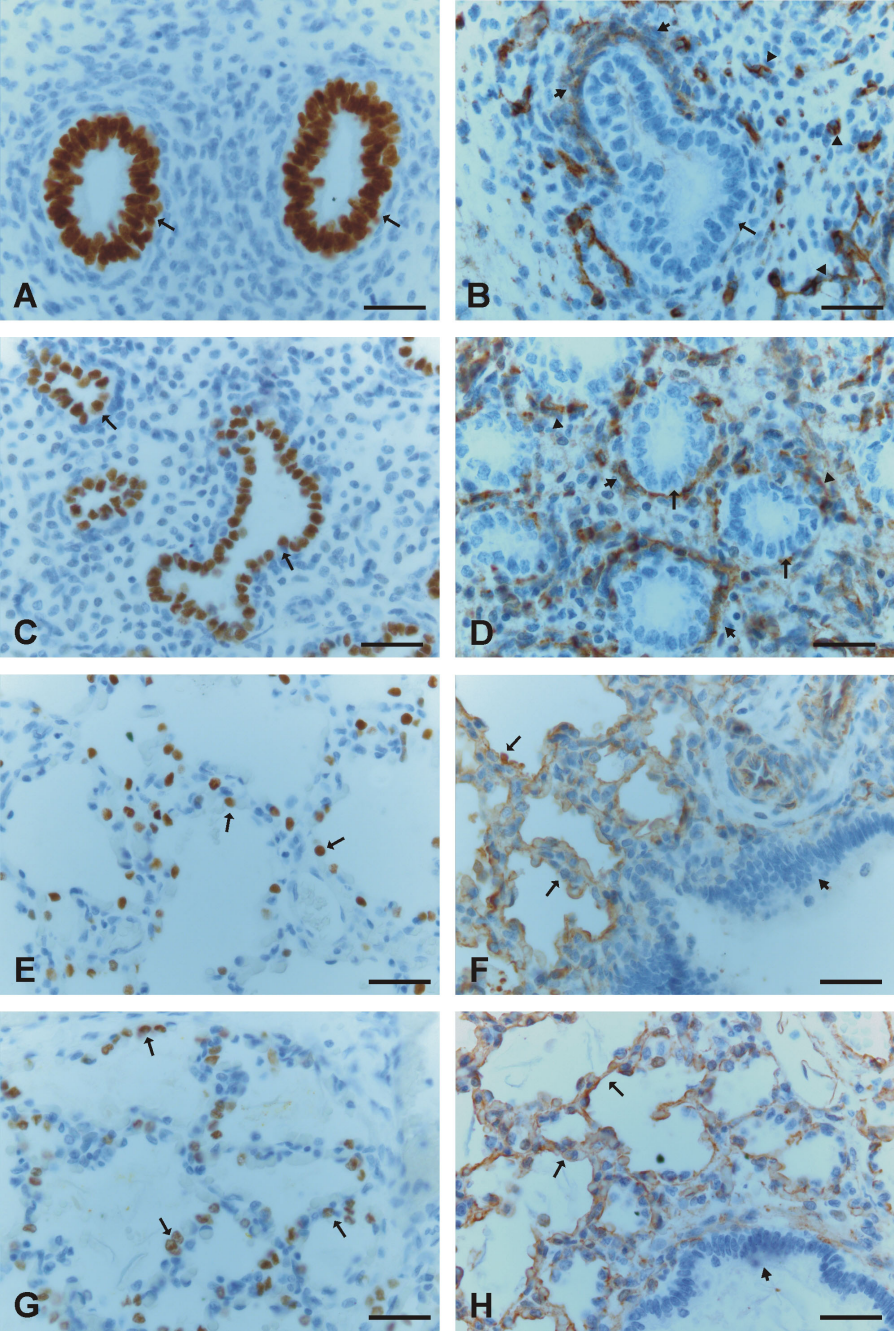
**Immunohistochemical stainings for TTF-1 and caveolin-1 during pseudoglandular, canalicular, saccular and alveolar periods of developing human lung**. 6A. Epithelial cells of small developing airways during pseudoglandular period are strongly positive for TTF-1 (arrows). 6B. Epithelial cells of a developing airway during pseudoglandular period are negative for caveolin-1 (arrow) while the cells surrounding the airway (short arrow) and obvious endothelial cells (arrowheads) are positive for it. 6C. Pretype II cells during the canalicular period are positive for TTF-1 (arrows). 6 D. Pretype II cells during the canalicular period do not show any positivity for caveolin-1 (arrows) while the cells rounding alveoli (short arrows) as well as the endothelial cells (arrowheads) are positively stained. 6E and 6G. Type II pneumocytes are positive for TTF-1 during saccular (6E) and alveolar (6G) periods (arrows). 6F and 6H. Caveolin-1 is positive during saccular (6F) and alveolar (6H) periods in alveolar epithelial cells (arrows) whereas bronchial epithelial cells do not exhibit any posivity (short arrows). Scale bars = 20 μm.

TTF-1 was expressed in all epithelial cells of developing airways (Figure 6A) while caveolin-1 was negative in airway epithelial cells but positive in stromal and endothelial cells (Figure 6B).

#### Canalicular period (weeks 16 to 28)

TTF-1 was faintly positive in occasional cells of bronchi while its expression was stronger especially in epithelial cells of terminal and respiratory bronchioles. TTF-1 was strongly expressed in alveolar epithelium i.e. in pretype II cells (Figure 6C). As in pseudoglandular period, caveolin-1 was negative in epithelial cells of alveoli and bronchioles but positive in stromal and endothelial cells (Figure 6D).

#### Saccular (weeks 28 to 36) period

TTF-1 was expressed in scattered epithelial cells of bronchi and bronchioles and it was clearly positive in alveoli in type II pneumocytes (Figure 6E). Caveolin-1 was positive in alveolar epithelial cells whereas bronchial epithelial cells did not exhibit any positivity (Figure 6F). Stromal and endothelial cells were also positive for caveolin-1.

#### Alveolar (weeks 36 to 40) period

TTF-1 was positive in scattered epithelial cells of bronchi and bronchioles and in alveolar type II pneumocytes (Figure 6G). Caveolin-1 staining was positive in alveolar epithelial, stromal and endothelial cells, but not in bronchial epithelium (Figure 6H).

### RT-PCR

Total RNA was isolated from paraffin embedded tissues and converted into cDNA for quantitative PCR analysis of E- and N-cadherin and β-catenin. First, the quality of synthesized cDNA was confirmed by GAPDH analysis. Due to the low expression of GAPDH, two samples from developing lung were excluded from the further analysis leaving 3 samples from the pseudoglandular period, 8 from canalicular, 7 from saccular and 3 from alveolar period. The analysis of the examined samples revealed that the relative expression of GAPDH was 1.02-fold (SD 0.15) during the pseudoglandular period, 1.17-fold (SD 0.31) during canalicular, 1.14-fold (SD 0.19) during saccular and 1.36-fold (SD 0.27) during the alveolar period compared to average adult lung. Since these differences were minor, GAPDH was considered as a house-keeping gene and used as the denominator in the quantification of E- and N-cadherin and β-catenin by RT-PCR.

**Figure 7:**
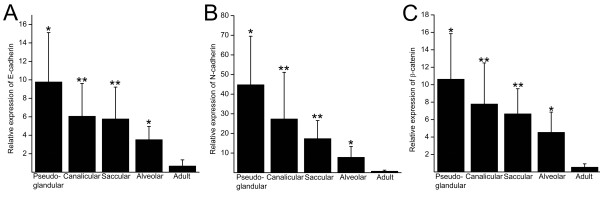
**Quantitative RT-PCR analysis of E-cadherin (A), N-cadherin (B) and β-catenin (C)**. The RNA was isolated from paraffin embedded tissue samples of pseudoglandular (n = 3), canalicular (n = 8), saccular (n = 7) and alveolar (n = 3) periods and from adult lung (n = 5). The isolated RNA was reverse transcribed into cDNA before real-time PCR. The results were analysed against the GAPDH expression to give relative expression values and related to one adult lung sample. One asterisk represents the probability value below 0.05 and two asterisks values below 0.01 compared to healthy control group.

E- and N-cadherin and β-catenin were all very highly expressed during the pseudoglandular period and their expression declined towards maturation (Figure 7). E-cadherin expression was 5.9-fold (SD 3.2) during the pseudoglandular period, 3.7-fold (SD 2.1) during canalicular, 3.4-fold (SD 2.1) during saccular and 2.1-fold during alveolar period as compared to average adult lung. N-cadherin was even more extensively upregulated, showing 32 (SD 18), 20 (SD 17), 12 (SD 6.6) and 5.6 (SD 3.9) times more expression than adult lung during the various developmental periods. β-catenin expression was 8.8-fold (SD 4.3) during pseudoglandular, 6.4-fold (SD 3.9) during canalicular, 5.5-fold (SD 2.4) during saccular and 3.8-fold (SD 1.9) during alveolar period compared to adult lung. However, as revealed by the the standard deviations, there was extensive variation between the samples in all analyzes and therefore the differences between the groups do not achieve statistical significance but the expression of both E- and N-cadherin and β-catenin in the developing lung differed significantly from the adult lung (p < 0,001).

## Discussion and Conclusions

The present study describes for the first time the immunohistochemical expression of E- and N-cadherin and β-catenin at the tissue level during human lung development at different gestational ages i.e. from week 12 to week 40 during the pseudoglandular, canalicular, saccular and alveolar periods. As anticipated, E-cadherin was found to be expressed throughout the various periods in both bronchiolar and alveolar epithelium. The expression of N-cadherin was also seen in epithelial cells of developing airways during all developmental stages, being at its strongest in the pseudoglandular and canalicular periods, though it remained positive in some scattered bronchial epithelial cells in later gestation i.e. during the saccular and alveolar periods. This finding was somewhat unexpected since traditionally N-cadherin has been assumed to be a marker of neural and mesenchymal cells. We have previously observed similar expression variability of several tight junctional proteins, namely claudins, in developing human lung [15]. β-catenin was widely expressed throughout all periods in various types of pulmonary cells including both epithelial, endothelial and mesenchymal cells, a not unexpected finding when taking into consideration its diverse roles in controlling cell biological functions and lung development. Not only the cell-specific localizations of β-catenin varied within lung tissue, but also its distribution within the cell i.e. during early lung development β-catenin was expressed mostly as nuclear type positivity within epithelial cells and also in cells underneath the epithelia of airways, whereas it was expressed mainly as epithelial membrane-bound positivity during mid- and late-gestation. In addition, immunohistochemical and RT-PCR findings of the developing lung were compared to that of the normal adult human lung. By both methods used, the expressions of N- and E-cadherin and β-catenin were shown to be higher during the lung development than in the adult lung.

In rat lung, it has been reported that all epithelial cells of the lung express both E- and P-cadherin during the early developmental stage whereas P-cadherin gradually disappears during development, initially from the main bronchi but eventually from all epithelial cells [19]. In the study of Kasper et al., localization of E-cadherin was examined in fetal human lung and it was noted that the epithelia of primary pulmonary primordium and the secondary bronchi were positive for E-cadherin with the immunoperoxidase and immunofluorescence techniques [20]. As far as we are aware there are no previous published studies on the immunohistochemical expression of E-cadherin in developing human lung which have investigated several gestational stages. The role of E-cadherin in the invasiveness of lung cancers and also its role as a commonly used epithelial cell marker in EMT-studies has attracted more attention in the previous studies [21,22]. In the present study we observed that in general E-cadherin was strongly expressed in the epithelia of bronchi and bronchioles from week 12 to week 40. It was also expressed in all epithelia of developing alveoli during the canalicular period when the cells that lines the alveoli are so called pretype II cells, which will subsequetly differentiate into type I and type II pneumocytes from weeks 24-28 onwards. E-cadherin was also clearly positive in type II pneumocytes during the saccular and alveolar periods, but its expression in type I pneumocytes was not clearly positive.

For identifying the phenotype of alveolar epithelial cells, we performed immunohistochemical stainings of our study material also for TTF-1 and caveolin-1. TTF-1 is known to be present in type II pneumocytes and also in subsets of bronchiolar cells, and moreover, the distribution of TTF-1 has been shown to follow patterns of distribution of surfactant protein-B in developing and diseased lungs [23,24]. TTF-1 has been detected in epithelial cells of human fetal lung as early as during the 11-12 weeks' gestation, a finding similar to ours [24]. Caveolins are the structural proteins that are necessary for the formation of caveolae membrane domains, and they are expressed in several types of organs and cells including type I pneumocytes [25]. In our study, E-cadherin was shown to be co-expressed with TTF-1 during various gestational ages. The positive expression for β-catenin was seen in cells that were positive either for TTF-1 or caveolin-1. During early gestation i.e. in pseudoglandular and canalicular periods N-cadherin was expressed in cells that seemed to be positive also for TTF-1.

There is little known about N-cadherin expression in the developing lung. Packer and colleagues evaluated the mRNA of N-cadherin in several mouse organs, and observed its expression in mesenchymal, but not in epithelial cells of lung [9]. N-cadherin has been assumed to be mainly expressed in mesenchymal cells, and thus has been considered as a marker of EMT, a structure postulated have a marked role also in the pathogenesis of lung cancers [26]. In our previous study, we observed that N-cadherin was expressed in a certain types of lung cancers such as endocrine tumors including small cell carcinomas and also in epithelial tumors like squamous cell carcinomas which are believed to originate from the bronchial epithelium [11]. We did not find any positivity for N-cadherin in normal or metaplastic bronchial epithelium in that particular study. In the present study, however, N-cadherin was clearly positive in the epithelia of developing bronchi and bronchioles during the early and mid gestation periods i.e. in the pseudo-glandular and canalicular periods, and it remained positive in some scattered bronchial cells also in later gestation i.e. in the saccular and alveolar periods. Alveolar cells, even pretype II cells, did not show any positivity for N-cadherin. The finding of the epithelial expression of N-cadherin during normal human lung development has two important implications. First N-cadherin might not be the most suitable marker for studying EMT, and secondly malignant cells may acquire the properties of fetal cells as indicated in the findings of the previous lung cancer studies.

β-catenin and the Wnt/β-catenin signaling pathway regulate certain aspects of branching morphogenesis, regional specialization of the epithelium and mesenchyme [13]. In an experimental study with mice, it was found that when the gene for β-catenin was excised in epithelial cells prior to embryonic day 14.5, the proximal lung tubules grew and differentiated normally, but the mice eventually succumbed due to respiratory failure [14]. This study also revealed that β-catenin staining in control animals as visualized by immunohistochemistry was present both as membrane-bound and also as nuclear positivity. These workers did not find nuclear staining in larger airways, when it was most prominent in epithelial cells lining the more peripheral tubules. We also observed that the immunohistochemical staining appeared mainly as two types; nuclear and membrane-bound. During early gestation i.e. in the pseudoglandular period, β-catenin expression in the epithelia of the smallest developing airways was mostly intensely nuclear, and also the mesenchymal cells beneath the airway epithelia displayed nuclear positivity. The epithelia of bronchi and largest bronchioles showed mainly membrane-bound positivity for β-catenin throughout all gestational periods. At the beginning of the canalicular period, the nuclear staining for β-catenin still existed, but it had eventually disappeared by the end of that period. β-catenin was also positive in pretype II cells and type II pneumocytes of alveoli, and also in endothelial and mesothelial cells. The results of our study support the results of the previous studies underlining the importance of β-catenin in the organogenesis of lung.

We concluded that E-cadherin was widely expressed in bronchial and alveolar epithelial cells throughout all developmental periods whereas N-cadherin showed epithelial positivity in bronchial epithelial cells mainly during the early developmental stages of lung. Thus it may be assumed that E-cadherin is relatively constantly present through ontogenesis of human lung whereas N-cadherin seems to be more restricted to certain developmental phases. The presence of β-catenin was observed in several cell types with distinct location in tissues and cells of various gestational stages indicating that this protein possesses several roles during lung development. The overall expression of the proteins and mRNAs of E- and N-cadherin and β-catenin were, however, higher in early compared to the situation in the later phases of gestation. Moreover, the expressions of these factors were higher during the lung development than in the adult human lung.

## Authors' contributions

RK participated in the design of the study, collected the study material and analyzed the immunohistochemical and clinical data of the patients. ELB participated in the design of the study, analyzed the immunohistochemical material and the design of the RT-PCR analyses. SL carried out the RT-PCR-analyses, analyzed the RT-PCR data and created the RT-PCR figures. All authors were involved in writing the paper and had final approval of the submitted and published versions.
